# Fibrosis Staging Using Direct Serum Biomarkers is Influenced by Hepatitis Activity Grading in Hepatitis C Virus Infection

**DOI:** 10.3390/jcm7090267

**Published:** 2018-09-11

**Authors:** Koji Fujita, Noriyuki Kuroda, Asahiro Morishita, Kyoko Oura, Tomoko Tadokoro, Takako Nomura, Hirohito Yoneyama, Takeshi Arai, Takashi Himoto, Seishiro Watanabe, Tsutomu Masaki

**Affiliations:** 1 Department of Gastroenterology and Neurology, Faculty of Medicine, Kagawa University, Miki 761-0701, Japan; 92m7v9@med.kagawa-u.ac.jp (K.F.); asahiro@med.kagawa-u.ac.jp (A.M.); kyoko_oura@med.kagawa-u.ac.jp (K.O.); t-nishioka@med.kagawa-u.ac.jp (T.T.); takako-n@med.kagawa-u.ac.jp (T.N.); hyoneyam@med.kagawa-u.ac.jp (H.Y.); seishirowww@yahoo.co.jp (S.W.); 2 Department of Clinical Laboratory, Kagawa University Hospital, Miki 761-0701, Japan; nkuroda@med.kagawa-u.ac.jp (N.K.); tarai@med.kagawa-u.ac.jp (T.A.); 3 Department of Medical Technology, Kagawa Prefectural University of Health Sciences, Takamatsu 761-0123, Japan; himoto@chs.pref.kagawa.jp

**Keywords:** liver cirrhosis, biomarkers, hepatitis C, chronic, biopsy, needle, interferons

## Abstract

Background: Chronic liver diseases (CLDs) generally progress from inflammation to fibrosis and finally to carcinogenesis. Staging of liver fibrosis progression is inevitable for the management of CLD patients. The purpose of this study was to compare the diagnostic abilities of Wisteria floribunda agglutinin-positive Mac-2 binding protein (WFA-M2BP), Enhanced liver fibrosis (ELF) score, Fibrosis-4 index, and AST to platelet ratio index (APRI) based on histopathological analysis of liver biopsy samples, from patients with positive Hepatitis C Virus (HCV) infection. Methods: Japanese patients with HCV infection who underwent liver biopsy examinations were enrolled in this study. WFA-M2BP levels and ELF scores were calculated using preserved serum samples. The fibrosis staging and activity grading were assessed using a modified METAVIR score. Results: A total of 122 patients were enrolled; the cohort included 27 patients with stage 1, 66 with stage 2, 20 with stage 3, and nine with stage 4 fibrosis. All four biomarkers distinguished stage 3 and stage 2 fibrosis. ROC curves revealed that all four fibrosis biomarkers presented AUC values greater than 0.8. Each of the four biomarkers in stage 2 was significantly different between the activity grade 1 and 2 groups. Conclusion: Fib-4 index and APRI were comparable with WFA-M2BP and ELF score in the diagnosis of advanced liver fibrosis in Japanese patients with HCV infection. All four biomarkers of liver fibrosis were influenced by histopathological activity grading, which implies that liver biopsy should be the gold standard to evaluate liver fibrosis staging even though several noninvasive biomarkers have been investigated well.

## 1. Introduction

Chronic liver diseases (CLDs) generally progress from inflammation to fibrosis and finally to carcinogenesis [1,2]. In addition to hepatocellular carcinoma, a couple of vital complications, such as chronic hepatic failure, rupture of esophagogastric varices, treatment-resistant ascites, and spontaneous bacterial peritonitis originate from cirrhosis, an end-stage liver fibrosis [3,4,5]. Furthermore, the liver fibrosis stage is partially reversed when therapeutic intervention removed the etiological source of the CLDs [6,7,8,9]. Therefore, staging liver fibrosis progression is inevitable for the management of CLD patients.

The gold standard for liver fibrosis staging might be liver biopsy, but the diagnostic accuracy of this technique is subject to sampling errors and observer variability [10,11]. Serious complications, such as bleeding, are well recognized. Therefore, noninvasive methods using blood samples or ultrasonographic examinations have been developed to determine liver fibrosis staging. Fibrosis-4 index (Fib-4 index) and AST to platelet ratio index (APRI) enable liver fibrosis staging using a routine blood examination for AST, ALT, and platelet count [12,13,14]. Fib-4 index and APRI are classified as ‘indirect markers’ because they reflect liver damage or a decline of liver function alone caused by liver fibrosis progression [15]. These indexes can identify advanced liver fibrosis [16].

Recently, another type of biomarker that assesses the status of hepatic fibrogenic mechanisms has been developed and is referred to as a direct biomarker. The two representatives of direct markers, Wisteria floribunda agglutinin-positive Mac-2 binding protein (WFA-M2BP) and enhanced liver fibrosis (ELF) score, are different from Fib-4 index and APRI in that WFA-M2BP and ELF score directly measure components of the extracellular matrix produced by activated stellate cells in fibrotic liver [17,18]. However, few studies have compared the diagnostic accuracy of WFA-M2BP and ELF score for liver fibrosis staging using liver histopathology as their study standard, although comparison of the two biomarkers using shear wave elastography as its study standard was recently reported [19].

The purpose of this study was to compare the diagnostic abilities of the two direct biomarkers, WFA-M2BP and ELF score, based on histopathological analysis of liver biopsy samples taken from Japanese patients positive for HCV infection.

## 2. Experimental Section

### 2.1. Patients

Japanese patients with HCV infection, who underwent percutaneous liver biopsy examinations in a clinical setting between May 1991 and July 1998, were enrolled. Patients who underwent interferon (IFN) therapy after liver biopsy examination were included, but patients who had hepatocellular carcinoma when the liver biopsy examination was performed were excluded.

### 2.2. Ethics

This study was conducted in accordance with the ethical principles of the Declaration of Helsinki and was approved by the Institutional Review Board at Kagawa University, Faculty of Medicine (approved number: Heisei27-096). When the current study was initiated, informed consent was obtained to allow for measurement of WFA-M2BP and ELF scores using the preserved serum samples. For patients who died and had no relatives listed in their clinical records, we provided opt-out methods for the relatives of the dead participants by publishing a summary of this study on our university website [20,21].

### 2.3. Clinical Data

The following clinical data were extracted from the participants’ medical records: age, gender, platelet count (Plt), aspartate aminotransferase (AST), alanine aminotransferase (ALT), total bilirubin (T-Bil), prothrombin time (PT), and serum albumin (Alb). WFA-M2BP was measured using a HISCL-2000i system (Sysmex, Kobe, Japan). The ELF score was calculated using an ADVIA Centaur XP system (Siemens Healthcare Diagnostics, Tarrytown, NY, USA). Serum hyaluronic acid, PIIINP, and TIMP-1 were measured and the score was calculated automatically using the following equation:

6.38−(ln (age) × 0.14) + (ln (HA) × 0.616 + (ln (PIIINP) × 0.586) + (ln (TIMP-1) × 0.472) [12]. The Fib-4 index was calculated using the following equation: age × AST (U/L)/(Plt (109/l) × √ALT (U/L)) [22]. Another index of liver fibrosis, APRI, was calculated using the following equation: 100 × (AST (U/L)/upper limit of normal AST values (U/L))/(Plt (109/L) [14]. In our hospital, 35 U/L was applied as the upper limit of the normal AST values. HCV infection was suspected by positive HCV antibody-II or III in sera and confirmed by polymerase chain reaction, combined reverse transcription-PCR, or branched chain DNA probe assay. For IFN therapy, nonpegylated IFNs, including natural IFN alpha and beta and recombinant IFN alpha-2a, alpha-2b, and beta, were employed. Sustained viral response (SVR) was defined as negative HCV DNA in sera 6 months after IFN therapy was completed.

### 2.4. Histopathological Analysis

The extent of fibrosis was assessed using a modified METAVIR score (modified from a past paper [23]) as follows: stage 1, portal or central fibrosis; stage 2, some septa; stage 3, many septa; stage 4, cirrhosis. Advanced fibrosis was defined as fibrosis stage 3 and 4. The METAVIR grading system was used to assess hepatic inflammatory activity [24]. Staging fibrosis and grading activity were performed by an experienced pathologist who specialized in liver pathology (S.W.).

### 2.5. Statistical Analysis

WFA-M2BP, ELF score, and other continuous or discrete variables, which were presented as the median and interquartile range, were analyzed using Mann–Whitney *U* test. Categorical variables were analyzed using Fisher’s exact test. *p* values less than 0.05 were considered statistically significant.

## 3. Results

### 3.1. Characteristics of the Patients

A total of 122 patients comprising 82 males and 40 females were enrolled in this study (Table 1). Based on the liver biopsy examinations, 27 patients had pointed out stage 1 fibrosis; 66 had stage 2 fibrosis; 20 had stage 3 fibrosis, and 9 had stage 4 fibrosis. The median values of age were not significantly different between the stage 1–2 group and stage 3–4 group. Other clinical data including grading of hepatitis activity, Plt, PT, total bilirubin, AST, ALT, and albumin are presented in Table 1.

### 3.2. Diagnosis of Advanced Liver Fibrosis Stage 3–4 by ROC Curve Analysis

**Figure 1:**
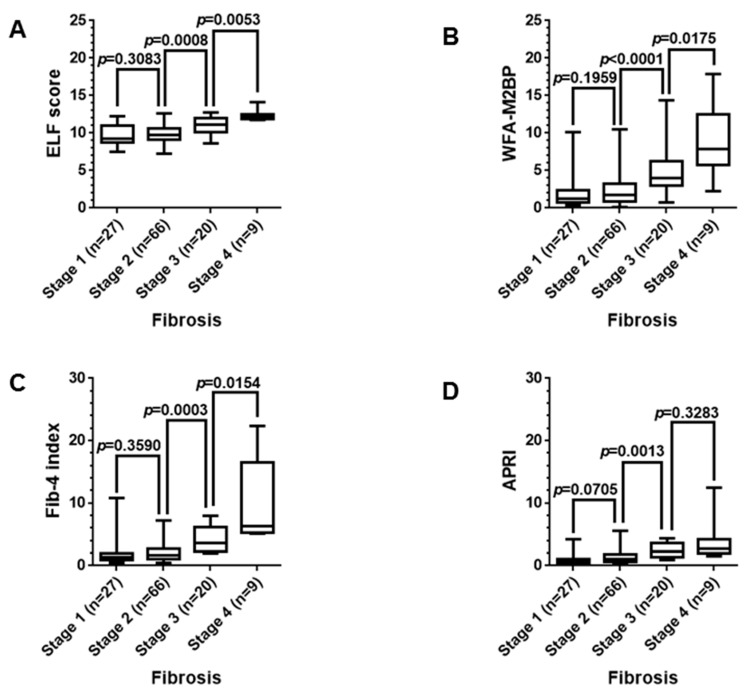
Difference in the median values for each fibrosis biomarker between two fibrosis stages. The four biomarkers distinguished stage 3 from stage 2 (*p* < 0.05); (**A**) ELF score, (**B**) WFA-M2BP, (**C**) Fib-4 index and (**D**) APRI. The median values of Wisteria floribunda agglutinin-positive Mac-2 binding protein (WFA-M2BP), Enhanced liver fibrosis (ELF) score, and Fibrosis-4 (Fib-4) index for stage 3 were also significantly different from those for stage 4. Data were analyzed using Mann–Whitney *U* test. *p* values less than 0.05 were considered statistically significant.

Differences in median values between two fibrosis stages were analyzed for each fibrosis biomarker using Mann–Whitney *U* test (Figure 1). As a result, all four biomarkers including WFA-M2BP, ELF score, Fib-4 index, and APRI failed to differentiate stage 2 from stage 1. However the four biomarkers distinguished stage 3 from stage 2. The median values of WFA-M2BP, ELF score and Fib-4 index for stage 3 were also significantly different from those for stage 4. The difference of APRI scores for stage 3 and 4 was not statistically significant.

**Figure 2:**
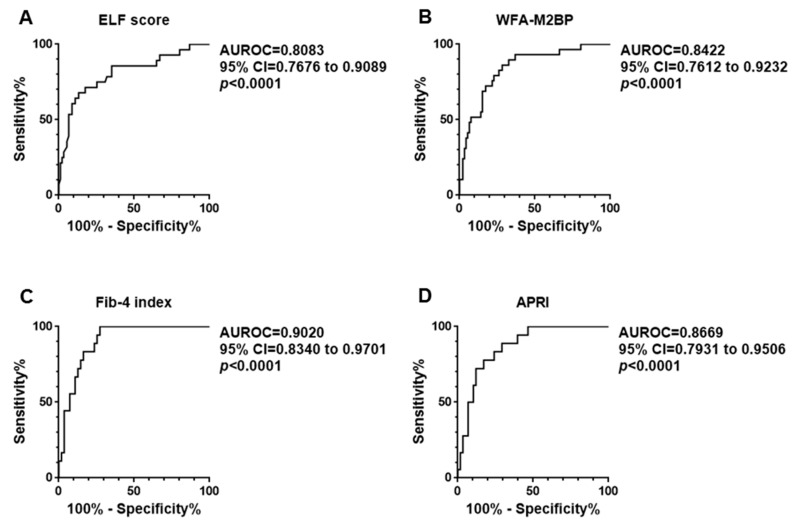
ROC analysis to assess the ability of the four fibrosis biomarkers to diagnose advanced liver fibrosis (stages 3 and 4) and distinguish these stages from nonadvanced stages 1 and 2; (**A**) ELF score, (**B**) WFA-M2BP, (**C**) Fib-4 index and (**D**) APRI. ROC curves revealed that all four fibrosis biomarkers presented AUC values greater than 0.8; the value for Fib-4 index was the highest among them (0.9020).

ROC analysis was performed to assess the ability of the four fibrosis biomarkers to diagnose advanced liver fibrosis (stages 3 and 4) and distinguish these stages from the nonadvanced stages 1 and 2 (Figure 2). ROC curves revealed that all four fibrosis biomarkers presented AUC values greater than 0.8; the value for Fib-4 index was the highest among them (0.9020). For the four fibrosis biomarkers, the sensitivity, specificity, and positive and negative likelihood ratios in the differential diagnosis of advanced liver fibrosis were also calculated using the median values and 75 percentile values of fibrosis biomarkers in the cohort as cut-off values (Table 2). When the median values were adopted as cut-off values, the WFA-M2BP and Fib-4 index presented greater sensitivities than those of ELF score and APRI. Negative likelihood ratios of WFA-M2BP and Fib-4 index resulted in less than 0.1, which means that the tests generate conclusive change from pretest to post-test probability to exclude advanced liver fibrosis [25]. When the 75 percentile values were employed as cut-off values, specificity of the four biomarkers were between 85% and 90%. Positive likelihood ratios of ELF score, Fib-4 index, and APRI surpassed 5, which indicate moderate increase in probability of advanced liver fibrosis [26]. WFA-M2BP showed positive likelihood ratio less than 5. According to the assessment above, an indirect serum biomarker, Fib-4 index, might be superior to direct biomarkers in determining advanced liver fibrosis, while Fib-4 index equaled to WFA-M2BP in excluding it.

### 3.3. Influence of Hepatitis Activity on Fibrosis Biomarkers

ELF score has been reported to be influenced by not only fibrosis staging but also activity grading of hepatitis. To assess the unreliability of the four fibrosis biomarkers to histopathological activity grading in liver biopsy specimens, 66 patients with fibrosis stage 2 were divided into 58 cases of grade 1 and eight cases of grade 2 based on a modified METAVIR score.

As shown in Table 3, all four biomarkers for the grade 2 group were significantly different from those for group 1. The median values and their interquartile ranges of four biomarkers were presented in Table 1. The median values of the grade 1 group were within the interquartile range of the fibrosis stage 1 group for all four biomarkers. The median values of the grade 2 group were within the interquartile range of the fibrosis stage 3 group for all four biomarkers as well. The data indicated that values of serum fibrosis biomarkers were variables of activity staging, suggesting that fibrosis staging by serum biomarkers has potential to overestimate or underestimate it.

Serum ALT levels were also significantly different between the grade 1 and 2 groups, suggesting that serum ALT level might provide a clue to estimate hepatitis activity grading. When a cut-off value of serum ALT level is set on the 75 percentile value of grade 1 group and the 25 percentile value for grade 2 group, serum ALT levels differentiate the grade 2 group from the grade 1 group in the fibrosis stage 2 group.

### 3.4. Prediction of Sustained Viral Response after Interferon Therapy

**Figure 3:**
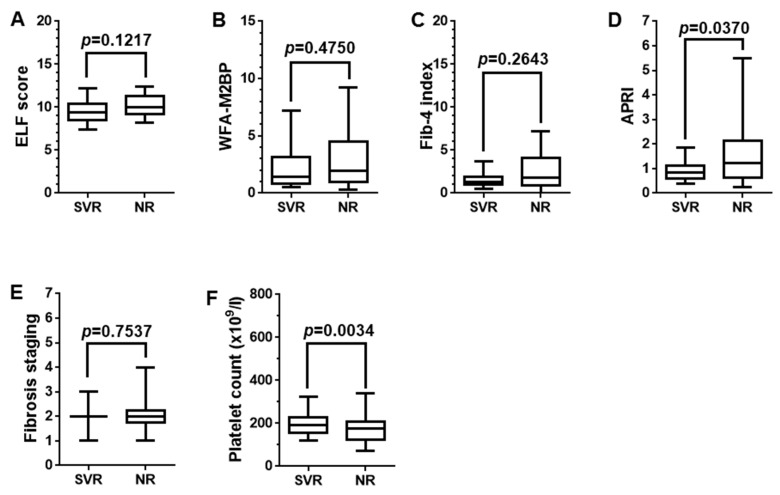
Prediction of sustained viral response after interferon therapy. (**A**–**D**) The median values of AST to platelet ratio index (APRI) alone were significantly different between the sustained viral response (SVR) group and the non-SVR group. (**E**) Histological staging of fibrosis was not significantly different between these two groups. (**F**) The results indicated that APRI was directly influenced by Plt in its equation and that the Plt of the SVR group was significantly greater than that of the non-SVR group. Data were analyzed using Mann–Whitney *U* test. *p* values less than 0.05 were considered statistically significant.

Among 122 patients, 47 underwent nonpegylated IFN therapy. As a result, 17 patients achieved SVR and 30 patients failed. Using the 47-case cohort who underwent IFN therapy after liver biopsy examination, predictive ability of the SVR was assessed in relation to the four fibrosis biomarkers. As shown in Figure 3, the median values of the APRI alone were significantly different between the SVR group and the non-SVR group (Figure 3D), though histological staging of fibrosis was not significantly different between two groups (Figure 3E). The results indicated that APRI was directly influenced by Plt in its equation and that the Plt of the SVR group was significantly greater than that of the non-SVR group (Figure 3F).

### 3.5. Examination Costs

Diagnostic values for advanced liver fibrosis were comparable among the four fibrosis biomarkers, as shown in the ROC curve analysis and the sensitivity and specificity analyses. From the perspective of examination costs in Japan, APRI, which costs 3.3 dollars, was the most inexpensive, followed by Fib-4 index, which costs 4.8 dollars. WFA-M2BP was approximately 4 times more expensive than Fib-4 index (16.9 dollars). Neither ELF score nor TIMP-1 is covered by insurance in Japan. ELF score might cost at least 28.2 dollars because the two biomarkers it uses, hyaluronic acid and PIIINP, currently cost a total of 28.2 dollars.

## 4. Discussion

The current study presented that (1) two emerging biomarkers, WFA-M2BP and ELF score, were equaled in staging liver fibrosis; (2) the ‘direct biomarkers’, WFA-M2BP and ELF score, were not necessarily superior to conventional ‘indirect biomarkers’, Fib-4 index and APRI, in a Japanese HCV-infected cohort; and (3) all four biomarkers of liver fibrosis presented variable values by histopathological activity grading, which implies that liver biopsy should be the gold standard to evaluate liver fibrosis staging even though several noninvasive biomarkers have been investigated well.

WFA-M2BP and ELF score are comparable in the diagnosis of advanced liver fibrosis as shown by Mann–Whitney *U* test in stage-by-stage analysis, ROC curve analysis, sensitivity, and specificity. The results followed the comparison of these two biomarkers based on fibrosis staging by transient elastography in a cohort comprised mainly of HBV-infected patients [27]. Contrary to recent work, neither WFA-M2BP nor ELF score were able to distinguish stage 2 from stage 1 in the current cohort, which might be due to the relatively smaller number of patients in the fibrosis stage 1 group 19. It remains unclear which patients are better suited to WFA-M2BP than ELF score or to ELF score than WFA-M2BP based on the data.

WFA-M2BP and ELF score, which are ‘direct biomarkers’, were not necessarily superior to conventional ‘indirect biomarkers’, Fib-4 index, and APRI, for identifying early or advanced liver fibrosis in the Japanese HCV-infected cohort as indicated by the current data. From the perspective of examination fees, indirect biomarkers should be superior to direct biomarkers, as recommended by the World health Organization [28]. However, the Plt in Fib-4 index and APRI equations might be influenced by a few of diseases encountered in daily practice, such as iron deficient anemia, drug-induced thrombocytopenia, and helicobacter pylori infection. WFA-M2BP and ELF score are more specific to the kinetics of liver fibrosis progression than are AST, ALT, and Plt because WFA-M2BP, hyaluronic acid, PIIINP, and TIMP-1 are secreted by the key player of liver fibrogenesis; activated hepatic stellate cells or myofibroblasts [29]. Supposing that direct biomarkers should not to surpass indirect biomarkers in staging liver fibrosis, predicting the potential of each biomarker for patient survival and hepatocarcinogenesis should be noted [30,31].

In the past report, ELF score was likely to incorrectly classify individuals [32]. Current data showed that all four biomarkers in the fibrosis stage 2 group presented a significantly greater median value in the activity grade 2 group than in the grade 1 group. The median value of the grade 2 group in the fibrosis stage 2 group can be interpreted as indicating fibrosis stage 3. It remains a problem in noninvasive liver fibrosis staging using serum samples to exclude interference from activity grading to fibrosis staging because no biomarkers for liver inflammation activity grading in serum have yet been established. However, our data proved that serum ALT levels differentiated the hepatitis activity grade 2 group from grade 1 group in the fibrosis stage 2 group. When appropriate cut-off values for serum ALT levels are established to speculate hepatitis activity in each fibrosis stage group, noninvasive staging of liver fibrosis should be more accurate. Because liver biopsy examinations in the current study were performed in a clinical setting, especially prior to starting interferon therapy in each patient, our cohort did not include much data of the fibrosis stage 1, 3, and 4 groups enough to present appropriate cut-off values of serum ALT levels for each fibrosis stage and activity grade.

Generally speaking, PLR more than 10 and NRL less than 0.1 significantly change the probability of disease 25. Our data showed that Fib-4 index and WFA-M2BP have potential to exclude advanced liver fibrosis, when cut-off values were set appropriately. Similar to it, Fib4-index, ELF score, and APRI were able to rule advanced liver fibrosis in based on alternative cut-off values. It is critical for accurate staging liver fibrosis using the serum biomarkers to increase pretest probability of advanced liver fibrosis because more noninvasive examination can be applied on much more patients than those who are assigned to liver biopsy examination, which might decrease the pre- and post-test probability for advanced liver fibrosis.

The limitations of the current study might lie in the fact that (1) the diagnostic accuracy of serum biomarkers was not compared to that of shear wave elastography, another emerging modality for liver fibrosis staging [33]; (2) one of the serum biomarkers, Fibro Test [34], was not included in the assessment; (3) the HCV genotype of was not determined in most of the patients; and (4) data concerning patients prognosis lacked in the study. Thus, how to choose a serum biomarker for a patient remains unclear.

In conclusion, the ‘direct biomarkers’, WFA-M2BP and ELF score, were not necessarily superior to conventional ‘indirect biomarkers’, Fib-4 index and APRI. All four biomarkers of liver fibrosis were influenced by histopathological activity grading, suggesting that liver biopsy should be the gold standard to evaluate liver fibrosis staging even though several noninvasive biomarkers have been investigated well.

## Figures and Tables

**Table 1:** Characteristics of the study population and their fibrosis markers.

Characteristics	Total	Stage 1 (*n* = 27)	Stage 2 (*n* = 66)	Stage 3 (*n* = 20)	Stage 4 (*n* = 9)	*p* (Stage 1–2 vs. 3–4)
Male/Female	82/40	17/10	47/19	14/6	4/5	0.5010
Age (year)	53 (42–59)	48 (36–60)	50 (40–58)	55 (49–59)	63 (55–64)	0.0201
ELF score	9.99 (9.14–11.27)	9.26 (8.73–11.00)	9.81 (9.10–10.61)	11.12 (10.13–12.03)	12.29 (11.82–12.46)	<0.0001
WFA-M2BP	2.25 (0.97–4.25)	1.26 (0.69–2.36)	1.81 (0.90–3.21)	4.03 (2.97–6.20)	7.86 (5.74–12.46)	<0.0001
Fib-4 index	1.79 (1.03–3.73)	1.95 (1.30–3.77)	1.58 (1.02–2.67)	3.57 (2.19–617)	5.99 (5.08–7.19)	<0.0001
APRI	1.13 (0.62–2.07)	0.63 (0.49–1.10)	1.10 (0.60–1.74)	2.18 (1.31–3.56)	2.69 (2.00–4.20)	<0.0001
Platelet count (10^9^/L))	178 (122–273)	154 (128–243)	183 (141–224)	134 (90–186)	81 (47–87)	<0.0001
PT (%)	85 (69–97)	86 (77–117)	92 (80–106)	76 (57–87)	76 (46–86)	0.0398
Total bilirubin (mg/dL)	0.8 (0.6–1.0)	0.8 (0.5–0.9)	0.7 (0.6–0.9)	1.1 (0.8–1.3)	1.5 (0.8–1.8)	<0.0001
AST (U/L)	64 (44–96)	36 (22–53)	60 (42–95)	73 (63–113)	74 (56–83)	0.0083
ALT (U/L)	98 (56–138)	32 (16–76)	103 (65–168)	101 (94–137)	63 (32–103)	0.9854
Albumin (mg/dL)	4.0 (3.7–4.2)	3.8 (3.5–4.2)	4.1 (3.8–4.3)	3.5 (3.3–4.0)	3.4 (3.0–3.7)	<0.0001

Continuous variables, which were presented as the median and interquartile range, were analyzed using Mann–Whitney U test. Categorical variables were analyzed using Fisher’s exact test. *p* values less than 0.05 were considered statistically significant.

**Table 2:** Diagnostic capability of fibrosis biomarkers.

	ELF Score	WFA-M2BP	Fib-4 Index	APRI
Cut off value = median value	9.97	2.19	1.76	1.13
Sensitivity (95% CI)	85.7 (67.3–96.0)	93.1 (77.2–99.2)	100 (81.5–100)	88.9 (65.3–98.6)
Specificity (95% CI)	61.5 (50.8–71.6)	63.0 (52.3–72.9)	63.6 (50.0–76.2)	62.1 (48.4–74.5)
PLR (95% CI)	2.17 (2.07–2.27)	2.57 (2.47–2.67)	2.75 (2.58–2.93)	2.34 (2.19–2.51)
NLR (95% CI)	0.24 (0.15–0.36)	0.08 (0.03–0.21)	0	0.18 (0.07–0.44)
Cut off value = 75 percentile value	11.27	4.25	3.73	2.07
Sensitivity (95% CI)	64.3 (44.1–81.4)	55.2 (35.7–73.6)	66.7 (41.0–86.7)	61.1 (35.8–82.7)
Specificity (95% CI)	87.9 (79.4–93.8)	85.9 (77.1–92.3)	89.1 (77.8–95.9)	87.9 (76.7–95.0)
PLR (95% CI)	5.31 (4.37–6.47)	3.90 (3.25–4.70)	6.11 (4.33–8.63)	5.06 (3.69–6.94)
NLR (95% CI)	0.24 (0.15–0.36)	0.52 (0.48–0.57)	0.37 (0.30–0.47)	0.44 (0.37–0.53)
Costs (dollars)	28.2	16.9	4.8	3.3

CI, confidence interval; PLR, positive likelihood ratio; NLR, negative likelihood ratio.

**Table 3:** Fibrosis staging using serum biomarkers is influenced by hepatitis activity grading.

	Grade 1 (*n* = 58)	Grade 2 (*n* = 8)	*p* (Grade 1 vs. 2)
ELF score	9.616 (9.037–10.38)	10.79 (10.64–11.74)	0.0001
WFA-M2BP	1.620 (0.870–2.875)	3.66 (2.37–4.29)	0.0050
Fib-4 index	1.433 (0.965–2.037)	4.374 (2.153–6.546)	0.0016
APRI	0.999 (0.596–1.418)	3.040 (1.478–4.47)	0.0023
ALT (U/l)	103 (58–133)	213 (133–308)	0.0037

Data were presented as the median and interquartile range (IQR) and analyzed using Mann–Whitney *U* test. *p* values less than 0.05 were considered statistically significant.

## Abbreviations

CLDs	Chronic liver diseases
CH	Chronic hepatitis
LC	Liver cirrhosis
HCC	Hepatocellular carcinoma
HBV	Hepatitis B virus
HCV	Hepatitis C virus
Fib-4 index	Fibrosis-4 index
APRI	AST to platelet ratio index
WFA-M2BPGi	Wisteria floribunda agglutinin-positive Mac-2 binding protein
ELF score	Enhanced liver fibrosis score
